# Qualitative evidence to improve guidelines and health decision-making

**DOI:** 10.2471/BLT.17.206540

**Published:** 2018-02-01

**Authors:** Etienne V Langlois, Özge Tunçalp, Susan L Norris, Ian Askew, Abdul Ghaffar

**Affiliations:** a Alliance for Health Policy and Systems Research, World Health Organization, avenue Appia 20, 1211 Geneva 27, Switzerland.; b Department of Reproductive Health and Research, World Health Organization, Geneva, Switzerland.; c Guidelines Review Committee Secretariat, World Health Organization, Geneva, Switzerland.

As governments are developing schemes for universal health coverage (UHC) and progressing towards the sustainable development goals (SDGs), they need relevant and context-sensitive evidence to support different policies and interventions. Decision-makers are increasingly using qualitative evidence to understand various socioeconomic contexts, health systems and communities. This type of evidence is useful to assess the needs, values, perceptions and experiences of stakeholders, including policymakers, providers, communities and patients, and is thus crucial for complex health decision-making. For instance, during the development of the World Health Organization (WHO) *Recommendations on antenatal care for a positive pregnancy experience*,^1^ qualitative evidence was used to understand what women want, need and value during pregnancy and antenatal care.^2^ The findings of two qualitative evidence syntheses helped to identify factors that influence access to antenatal care for women^3^ and provision of good-quality services by health-care providers.^4^ The findings also contributed to informing the acceptability of the recommendations and their implementation considerations.

Qualitative evidence syntheses, which combine and analyse evidence from individual qualitative studies, have emerged as a key approach to inform guideline development and address implementation considerations in diverse country settings and complex health systems.^5^ By extending beyond the evidence on benefits and harms provided by effectiveness reviews, qualitative evidence syntheses clarify the interplay between stakeholders, health systems and context. They are fundamental to understand the values and preferences of end-users, to assess the acceptability and feasibility of health and social interventions, and to explore the effects of different interventions on equity.^5^ WHO, as a producer of clinical, public health and health system guidelines, increasingly uses qualitative evidence syntheses to ensure that its recommendations reflect the needs of its audience and the varied contexts where recommended interventions will be implemented.

Beyond global guidance, qualitative evidence is invaluable for national and local decision-makers and practitioners to understand factors influencing the implementation and scale-up of health policies and programmes. For instance, policy-makers require evidence on factors that influence vaccination coverage, satisfaction and retention of health-care workers or quality of care in health facilities. Qualitative data have proven essential in planning, developing and implementing health policies and interventions, including in low- and middle-income countries, for example to prevent and treat malaria during pregnancy^6^ or to promote respectful maternity care in country programmes.^7^ Qualitative evidence also helps policy-makers and programme managers to make decisions about how to adapt a given WHO guideline and how to prioritize specific recommendations for implementation.

Yet, despite the relevance of findings from qualitative evidence syntheses, there is limited guidance on how to assess and use this evidence in policy and practice. To address this need, the Alliance for Health Policy and Systems Research and the WHO Department of Reproductive Health and Research have supported the development of a new approach called GRADE-CERQual (Grading of Recommendations Assessment, Development and Evaluation-Confidence in the evidence from reviews of qualitative research). The GRADE-CERQual approach is used to describe how much confidence decision-makers and other users can place in findings from qualitative evidence syntheses by transparently assessing methodological limitations, coherence, adequacy and relevance.^8^^,^^9^ The approach is similar to GRADE, which is widely used to assess how much confidence to place in review findings on the effectiveness of health interventions.

New guidance on how to apply the GRADE-CERQual approach is now available as a special series of articles to support stakeholders conducting reviews of qualitative research and using their findings to inform decision-making.^8^ The articles explain the approach step-by-step and why and how the approach was developed. This guidance also provides information on how to make an overall assessment of confidence and how to present key findings and confidence assessments.

The GRADE-CERQual approach was developed as a global public good to advance research methods and promote the uptake of qualitative findings in decision-making within and beyond the health sector. This guidance is also aligned with a global movement towards the generation and use of a wide array of evidence in policy-making.^10^^,^^11^ Finally, this approach is important to better understand complex policies and programmes across contexts and to inform system-wide interventions relevant to UHC and the SDGs. As such, WHO aims to innovate and test methods to improve the development, adaptation and use of its guidelines, and to support countries towards greater use of evidence in health decision-making.
